# Task shifting of cardiovascular risk assessment and communication by nurses for primary and secondary prevention of cardiovascular diseases in a tertiary health care setting of Northern India

**DOI:** 10.1186/s12913-019-4864-9

**Published:** 2020-01-03

**Authors:** Kavita, J. S. Thakur, R. Vijayvergiya, S. Ghai

**Affiliations:** 1 National Institute of Nursing Education Post Graduate Institute of Medical Education and Research (PGIMER), Chandigarh, India; 2 Department of Community medicine and School of Public Health, PGIMER, Chandigarh, India; 3 Department of Cardiology, PGIMER, Chandigarh, India

**Keywords:** Cardiovascular risk assessment, WHO/ISH risk prediction charts, Task shifting, Risk communication, Primary prevention, Secondary prevention, Medication adherence

## Abstract

**Background:**

Cardiovascular diseases (CVDs) are the leading cause of morbidity and mortality in India. CVDs are to a large extent preventable with the availability of wide range of interventions focusing on primary and secondary prevention. However human resource deficit is the biggest challenge for implementing these prevention programs. Task shifting of the cardiovascular risk assessment and communication to nurses can be one of the most viable and sustainable option to run prevention programs.

**Methods:**

The study was quasi experimental in nature with 1 year follow up to determine the effect of CVD risk assessment and communication by nurses with the help of risk communication package on primary and secondary prevention of CVDs. The study was done in the outpatient departments of a tertiary health care center of Northern India. All the nurses (*n* = 16) working in selected OPDs were trained in CVD risk assessment and communication of risk to the patients. A total of 402 patients aged 40 years and above with hypertension (HTN) were recruited for primary prevention of CVDs from medicine and allied OPDs, whereas 500 patients who had undergone CABG/PTCA were recruited from cardiology OPDs for secondary prevention of CVDs and were randomized to intervention (*n* = 250) and comparison group (*n* = 250) by using block randomization. CVD risk modification and medication adherence were the outcomes of interest for primary and secondary prevention of CVDs respectively.

**Results:**

The results revealed high level of agreement (k = 0.84) between the risk scores generated by nurses with that of investigator. In the primary prevention group, there were significantly higher proportion of participants in the low risk category (70%) as compared to baseline assessment (60.6%) at 1 year follow up. Whereas in secondary prevention group the mean medication adherence score among intervention group participants (7.60) was significantly higher than that of the comparison group (5.96) with a large effect size of 1.1.(*p* < 0.01).

**Conclusion:**

Nurse led intervention was effective in risk modification and improving medication adherence among subjects for primary and secondary prevention of CVDs respectively.

**Trial registration:**

Trial registration no CTRI/2018/01/011372 [Registered on: 16/01/2018] Trial Registered Retrospectively.

## Background

Non communicable diseases (NCDs) are the leading cause of death globally. In 2012 there were 56 million total global deaths, out of which nearly two third i.e. 68% (38 million) were due to NCDs. Four major diseases were responsible for 82% of NCDs deaths. Nearly half (46.2%) i.e. 17.5 million of these deaths were due to cardiovascular diseases. Cancer contributed about 21.7% (8.2 million), respiratory diseases 10.7% (4.0 million) and diabetes 4% (1.5 million) [1]. CVDs are also among the top killer in South –East Asia region (SEAR) [2]. In India nearly one fourth (26%) of the total deaths were due to CVDs in the year 2012 [3].

The rising burden of NCDs, specifically cardiovascular diseases (CVDs) is putting a huge demand on existing health care resources including human resource. Evidence from some of the well known community trials in different parts of the world revealed that CVDs are to a great extent preventable with the reduction in their risk factors [4–9]. CVDs, primarily coronary artery disease is associated with a number of risk factors which are largely preventable with various primary and secondary prevention strategies [10].

Primary prevention strategies/programs target individuals with risk factors for CVDs. In order to implement these strategies identification of high risk individuals is the first step which is followed by risk reduction strategies [11]. Whereas secondary prevention strategies/programs on the other hand are for those patients who have already suffered a cardiovascular event e.g. heart attack or stroke. It primarily includes lifestyle modification and treatment adherence.

However to successfully implement these prevention programs, availability of trained health manpower is an essential prerequisite. India like many other Low and middle income countries (LMICs) faces the shortage of Human resource for health (HRH) /health manpower [12]. Thus implementing CVD prevention program with the existing scarce human resources is a real challenge [13]. Although training and recruiting more manpower can be a long term solution to address this issue, however task shifting can be thought of as one of the most sustainable option to meet immediate needs. World Health Organization defines Task shifting as transferring of clinical tasks from physicians to trained non physician health workers (NPHW) [14]. It has been proven as an effective, successful and cost effective method in reducing the global CVD epidemic in low and middle income countries [15, 16]. Task shifting is recommended at different levels with different categories of health workforce. Nurses are ideal choice for the task shifting of CVD risk assessment and communication as they are trained health care professionals. Role of nurses in CVD risk assessment and management is well established in different parts of the World [17–22].H.owever authors could not find any evidence related to the task shifting interventions for CVD risk assessment and communication by professional nurses from India. So this study was undertaken to evaluate the task shifting approach of cardiovascular risk assessment and communication by nurses working in a tertiary health care hospital of Northern India .

## Methods

The study was quasi experimental in nature to determine the effect of CVD risk assessment and communication by nurses on primary and secondary prevention of CVDs. CVD in the present study refers to coronary artery disease. The study was conducted in the out patient departments (OPDs) of a tertiary health care hospital in Chandigarh (North India). All the nurses (*n* = 16) working in the selected OPDs of the hospital were recruited for the study. After obtaining consent, these nurses were trained to calculate 10 year absolute risk of CVDs with WHO/ISH risk prediction charts and to communicate risk as well as to counsel subjects for risk reduction strategies. Risk communication package was developed by investigator to train nurses in cardiovascular risk assessment and communication. Investigator in the study is a qualified registered nurse, who was trained and certified as competent in CVD risk assessment and communication by a faculty physician (JST).

Validation of the intervention package was done by 11 experts from the field of: Cardiology (*n* = 2), community medicine (*n* = 4), nursing (*n* = 4) and fine arts (*n* = 1) It consisted of booklet for nurses, patient education booklet and flash cards for patient education. Nurses were trained by using this package and the duration of training was 6–8 h (as recommended in WHO training manual) [23, 24]. Training methodology included lectures, group work, role plays, case scenarios and interactive sessions. Each lecture was followed by a practical session and group work. On site refresher training (approx. 2 h) was also done as required. Nurses were subsequently given a certificate for training and participation in the study.

These trained nurses after successfully completing the training recruited patients from the OPDs of a tertiary health care hospital. Twelve nurses recruited the patients for primary prevention of CVDs from medicine and allied OPDs whereas four nurses enrolled subjects from cardiology OPDs for secondary prevention of CVDs. Each trained nurse recruited minimum of 20–25 patients.

For primary prevention of CVDs, 402 patients aged 40 years and above with hypertension were included in the study. Sample size was calculated based on the prevalence of hypertension in Chandigarh (50%) [25] at 95% confidence interval, 80% power and assuming 10% attrition. Patients were screened for hypertension by measuring blood pressure and those with the history of hypertension or who were found hypertensive on screening were included in the study. Written informed consent was obtained from all the participants. Patients with the history of any fatal or nonfatal cardiovascular event were excluded in the primary prevention group.

Blood pressure was recorded in sitting position in the left arm after the participant had been seated for 5 min to the nearest 1 mmHg using aneroid blood pressure measuring device. Two readings were taken and their mean was used for analysis. Hypertension was diagnosed based on JNC criteria i.e. past medical history or if the systolic BP was ≥140 mmHg or diastolic BP ≥90 mmHg. Diabetes screening was done by assessing random blood sugar (RBS) using freestyle optium glucometer. A person was considered to be diabetic if he/she was on treatment (insulin/oral hypoglycaemic agents) for diabetes or had RBS ≥ 200 mg/dl as per National Programme for Prevention and Control of Cancer Diabetes Cardiovascular Disease and Stroke (NPCDCS) guidelines [26]. All current smokers and those who had quit smoking < 1 year before the assessment were considered smokers. Fagerstrom test for nicotine dependence (FTND) [27] was used to assess their dependence level. CVD risk was assessed using World Health Organization /International Society for Hypertension (WHO/ISH) risk prediction charts (without cholesterol) for SEAR D region.

These charts predict the absolute risk of fatal or nonfatal cardiovascular event in the next ten years by using five individual risk factors i.e. age, gender, systolic blood pressure, smoking status and presence or absence of diabetes. The risk level were classified as <10% (low risk), 10 to <20% (moderate risk), 20 to <30% (high risk) and > 30% (very high risk) [28, 29].

Risk assessment was followed by risk communication and counselling for risk reduction by trained nurses with the help of risk communication package. All the nurses were given standardized risk communication material which included booklet for nurses, flash cards and patient education booklets. However the communication script was not standardized keeping in mind the needs of individual patients.

After the explanation of the risk, subjects were asked about the understanding of the same which was evaluated using a checklist. After this initial visit there were three telephonic follow ups (at 1st, 3rd and 6th month) and one face to face follow up at 1 year. There was no control arm in the primary prevention group and intervention was given to all the subjects. For establishing the reliability of CVD risk assessment done by nurses, investigator evaluated all the risk assessments and interrater reliability was calculated by using Kappa statistic.

Risk communication by nurses was evaluated by using standardized Gap Kalamazoo communication skill assessment form (GKCSAF) [30]. GKCSAF has nine essential communication elements rated on a 5 point Likert Scale (1 = Poor, 2 = Fair, 3 = Good, 4 = Very good, 5 = Excellent). The score on GKCSAF ranges from 9 to 45 and the score of 27 and above is considered to be an evidence of good communication skills [31]. Investigator evaluated all the risk communication by nurses and one third of them were also assessed by external rater to establish the reliability of invesigator’s scores. External rater involved in the study were either STI counsellor or nursing faculty or a Ph.D scholar. Intra class coefficient correlation was used to calculate interrater reliability.

**Fig. 1:**
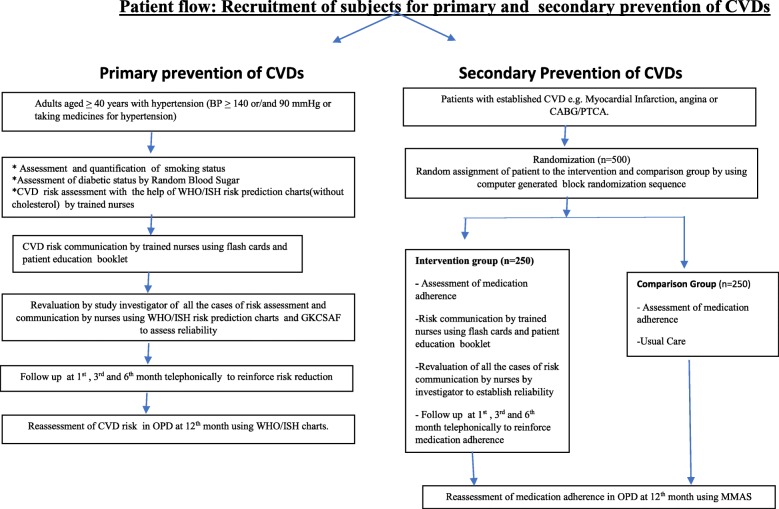
Patient flow: Recruitment of subjects for primary and secondary preventiton of CVDsfor primary prevention of CVDs

For secondary prevention of CVDs, a total of 500 patients with coronary artery disease who had undergone PTCA (percutaneous transluminal coronary angioplasty) /CABG (coronary artery bypass graft surgery) were randomized to intervention (*n* = 250) and comparison (*n* = 250) group by using computer generated block randomized sequence. The sample size calculations were based on the prevalent medication adherence rate of 50% [32] among CAD patients to the desired 80%. All the calculations were done for 80% power and 95% confidence level. Intervention in this group included risk communication and counselling about lifestyle modification by trained nurses or investigator (in case of non availability of trained nurse) only in the intervention group. Risk assessment was not required in these individuals as they were already in high risk category due to the presence of coronary artery disease. Initial risk communication was followed by, three telephonic follow ups at 1st, 3rd and 6th months to reinforce medication adherence and lifestyle modification. Comparison group received the usual care. Medication adherence was the outcome of interest for which a validated Hindi version of eight item Morisky Medication Adherence Scale (MMAS) [33] was used. The last follow up at 1 year was done face to face in both the groups to assess the effect of intervention on medication adherence. The recruitment process for the study is depicted in the flow diagram in Fig. 1.

Data entry and analysis was done using SPSS 19 software. Descriptive data was presented as measure of central tendency and dispersion. t-test and Mc Nemar tests were used to assess the effect of intervention on CVD risk modification and medication adherence in primary and secondary prevention group respectively. Cohen’s d was used as a measure of effect size and interpreted as small (0.2), medium (0.5) and large (0.8) effect size.

Kappa statistic and intraclass correlation was used to assess the reliability of risk assessment and risk communication by nurses. All tests were done at 0.05 level of significance.

The study protocol was reviewed and approved by the Institutional Ethics Committee of Post graduate institute of medical education and research (PGIMER), Chandigarh. Written informed consent was taken from both nurses and patients prior to enrolment. The trial is registered in the clinical trial registry of India.(registration no CTRI/2018/01/011372) . Detailed methodology is published elsewhere [34].

## Results

All the nurses (*n* = 16) in the study were female with the mean age of 38 ± 9.2 years. As regard professional qualification three fourth (75%) of them were graduate, 6.2% postgraduate and 18.8% were diploma holders. Out of the total 16 nurses, twelve were working in medicine and allied OPDs who recruited patients for primary prevention whereas four nurses from cardiology OPDs recruited patients for secondary prevention of CVDs. A total of 402 patients were recruited from medicine and allied OPDs for primary prevention of CVDs by a trained nurse/ investigator. Nurses carried out intervention of risk assessment and communication in about two third (68.6%) of the total patients in primary prevention group. Intervention in the remaining one third was done by investigator due to unavailability of trained nurses. Since nurses and investigator had similar findings so the results were pooled together Results showed that nurses performed the task of CVD risk assessment with high degree of reliability as the interrater reliability of risk assessment by trained nurses and investigator was 0.84(Kappa statistic) that means high level of agreement.

Risk assessment was followed by risk communication. All the nurses demonstrated good communication skills as evident from the mean communication score range of 29.7 to 35.8as per the GKCSAF.

Intraclass coefficient correlation (ICC) was used to assess interrater reliability of risk communication between investigator and external rater scores. Results demonstrated high interrater reliability with overall ICC of 0.97. The socio demographic profile of the participants in the primary prevention group revealed that 54% were males. Proportionately more males (41%) were in age group 60-69and married (93.1%). Nearly one third of the males (32.3%) and females (36.3%) belonged to the lower middle class (Table 1). A total of 360 (89.5%) subjects were available for final follow up at 1 year in primary prevention group. However for telephonic follow ups 99.7, 98.5 and 94.5% subjects were available at 1st, 3rd and 6th month follow up respectively Six subjects (1.5%) died of all cause mortality during the course of the study. Mean duration of follow up was 12.06 ± 0.20 months.

**Table 1:** Socio demographic profile of subjects enrolled for primary prevention of CVDs

S.No	Variable	Male *n* = 217	Female *n* = 185	Total *N* = 402	*X*^*2*^	*P* value
1.	Age group					
	40–49	27 (12.4)	45 (24.3)	72 (17.9)		
	50–59	76 (35.0)	71 (38.4)	147 (36.6)	13.27	.004
	60–69	89 (41.0)	54 (29.2)	143((35.6)		
	≥ 70	25 (11.5)	15 (8.1)	40 (10)		
2.	Marital Status					
	Never married	–0	1 (0.5)	1 (0.2)		
	Currently married	202 (93.1)	132 (71.4)	334 (83.1)	33.97	.001
	Separated	0	1 (0.5)	1 (0.2)		
	Widowed	15 (6.9)	51 (27.6)	66 (16.4)		
3.	Family Type					
	Nuclear	99 (45.6)	83 (44.9)	182 (45.3)	2.36	.307
	Joint	118 (54.4)	100 (54)	218 (54.2)		
	Others	0	2 (1.1)	2 (0.5)		
4.	Socioeconomic class (Kuppuswamy)					
	Upper (I)	3(1.4)	7 (3.8)	10 (2.5)	5.71	0.22
	Upper Middle (II)	109(50.2)	77 (41.6)	186 (46.3)		
	Lower middle (III)	70(32.3)	67 (36.2)	137 (34.1)		
	Upper Lower (IV)	35(16.1)	33 (17.8)	68 (16.9)		
	Lower (V)	0	1 (0.5)	1 (0.2)		

Figures in parentheses are percentages.

Significant reduction was recorded in CVD risk factors among both males and females at 1 year follow up. Mean SBP decreased from140.94 mmHg to 128.16 mmHg and 136.57 mmHg to 125.91 mmHg with the mean difference of − 12.78 mmHg and − 10.66 mmHg in males and females respectively. Whereas mean diastolic blood pressure decreased from 88.49 mmHg to 82.60 mmHg in males and 85.21 mmHg to 80.48 mmHg in females with the mean difference of − 5.88 and − 4.72 respectively. Significant reductions were also found in mean random blood sugar levels (*p* < .01). Cohen’s d was calculated as a measure of effect size (ES), which showed that systolic blood pressure has large effect size (0.76), DBP moderate ES (0.51) and RBS had low ES (0.2). Mean % change was −6.18 for DBP and − 8.52 for SBP. Mean FTND score also significantly decreased from 5.35 at baseline to 2.64 at 1 year follow up (*p* < 0.01). Change in FTND score showed a large effect size with the Cohen’s d of 1.2(Table 2).

**Table 2:** Mean change in CVD risk factors among subjects enrolled for primary prevention of CVDs at 1 year follow up

S.No	CVD risk factor	N	Baseline	Post intervention	Mean change (95% CI)	% Change	t statistic	P valve	Cohen’s d
			Mean ± 1 SD	Mean ± 1SD					
1.	Systolic BP (mmHg)								
	Male	201	140.94 ± 17.51	128.16 ± 14.28	−12.78 (−15.41, −10.14)	−9.06	9.55	.01	0.79
	Female	159	136.57 ± 17.00	125.91 ± 11.83	−10.66 (−13.14,-8.18)	−7.80	8.49	.01	0.72
	Total	360	139.01 ± 17.40	127.16 ± 13.29	−11.84 (−13.67, −10.01)	−8.52	11.68	.01	0.76
2	Diastolic BP (mmHg)								
	Male	201	88.49 ± 12.52	82.60 ± 9.16	−5.88 (−7.71,-4.05)	−6.65	6.33	.01	0.53
	Female	159	85.21 ± 10.29	80.48 ± 8.56	−4.72 (−6.48,-2.97)	−5.55	5.31	.01	0.49
	Total	360	87.04 ± 11.69	81.66 ± 8.95	−5.38 (−6.65, −4.09)	− 6.18	7.52	.01	0.51
3.	RBS (mg/dl)								
	Male	148	139.16 ± 66.31	128.42 ± 42.93	−10.73 (−20.47,-1.00)	−7.71	2.18	.03	0.19
	Female	116	148.73 ± 66.57	135.38 ± 47.10	−13.34 (−24.25,-2.43)	−8.97	2.42	.01	0.23
	Total	264	143.36 ± 66.47	131.48 ± 44.86	−11.88 (−19.10, −4.66,)	−8.28	2.68	.01	0.20
4.	FTND score	28	5.35 ± 2.46	2.64 ± 1.98	2.71 (1.89,3.53)	50.65	6.82	<.01	1.2

Cohen’s d: small (0.2), medium (0.5) and large (0.8)

**Fig. 2:**
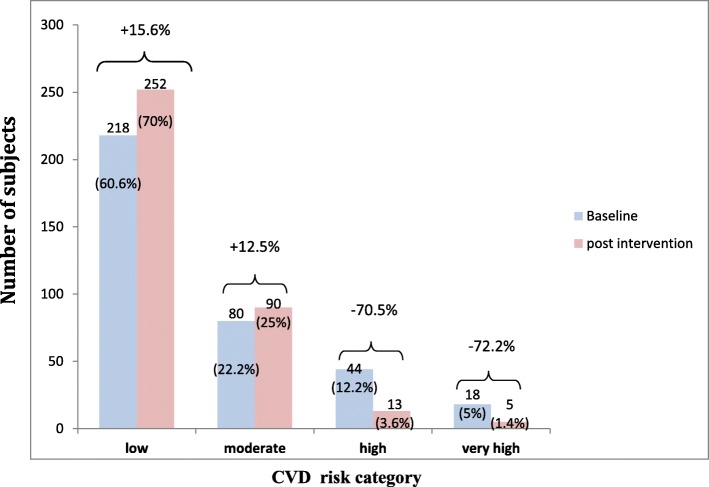
Shift in the WHO/ISH risk category baseline vs postintervention among subjects enrolled for primary prevention of CVDs at 1 year follow up in a tertiary health care hospital in Chandigarh (*N* = 360)

Shift in the CVD risk category as per WHO/ISH risk prediction charts was observed among subjects at 1 year follow up (Fig. 2). Proportion of participant in the low risk category increased from 60.6% at baseline to 70% at 1 year follow up with the percentage increase of 15.6%. However there was slight increase in the proportion of participants in moderate risk category (25%) at 1 year as compared to baseline (22.2%) because of the shift of participant from higher risk categories. Whereas number of participants in the high and very high risk category decreased after the intervention because of the shift to the lower risk categories.

**Fig. 3:**
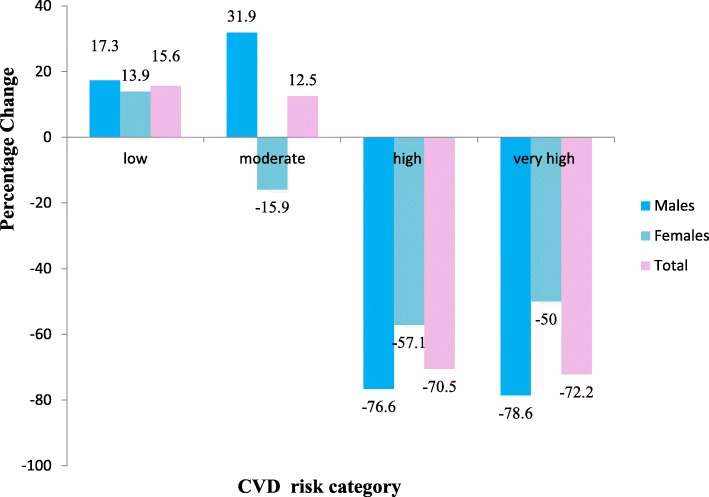
Percentage change from baseline to postintervention in WHO/ISH risk category among males and females enrolled for primary prevention of CVDs in a tertiary health care hospital in Chandigarh

Sexwise distribution of percentage change in WHO/ISH risk category revealed that in the low risk category males (17.3%) had larger percentage increase as compared to the females (13.9%). Whereas in the moderate risk category there was a percentage decrease (− 15.9%) for females but with the overall percentage increase of 12.5% for both sexes. In high and very high risk category the percentage decrease was − 76.6%and − 78.6% for males and − 57.1% and − 50% for females respectively (Fig. 3).

Majority of the subjects (92.7%) in the low risk category remained in the same risk category at 1 year follow up. Among subjects in the moderate risk category nearly two third (61.3%) remained in the same category, however approximately one third (35%) moved to the low risk category. In the high risk group 36.4% subjects shifted down two categories i.e. from high to low and 45.5% subjects shifted one category from high to moderate. There were significantly higher proportion of participants in the low risk category and lower proportion in high risk category at 1 year follow up in comparison to baseline assessment (Fig. 2).

A total of 500 patients were recruited for secondary prevention of CVDs. Four nurses communicated the risk in these patients. Intraclass coefficient correlation was calculated for assessing interrater reliability which demonstrated high interrater reliability with overall ICC of 0.94. Mean communication score of nurses ranged from 31.4 to 37.3. All the nurses enrolled for secondary prevention demonstrated good communication skills while communicating risk to patients.

Socio demographic profile of patients (*n* = 500) enrolled for secondary prevention of CVDs revealed that majority of participants in both intervention (84%) and comparison group (82%) were males. Groups were comparable in all socio demographic variables (age, sex, marital status, family type, socioeconomic status, medication adherence, number of medicines, family history and duration of treatment, (Table 3).

**Table 3:** Sociodemographic profile, baseline medication adherence, number of prescribed medication and family history of early CVD among subjects enrolled for secondary prevention of CVDs

S.No	Variable	Intervention group (*n* = 250)	Comparison Group (*n* = 250)	Total N-500	*X*^*2*^	*P* value
1.	Sex					
	Male	210 (84)	205 (82)	415 (83)	0.35	0.55
	Female	40 (16)	45 (18)	85 (17)		
2.	Age					
	30–39	8 (3.2)	7 (2.8)	15 (3)		
	40–49	40 (16)	32 (12.8)	72 (14.4)	2.38	0.66
	50–59	80 (32)	80 (32)	160 (32)		
	60–69	86 (34.4)	100 (40)	186 (37.2)		
	≥ 70	36 (14.4)	31 (12.4)	67 (13.4)		
3.	Marital Status					
	Never married	0	1 (0.4)	1 (0.2)	3.05	0.38
	Currently married	228 (91.2)	224 (89.6)	452 (90.4)		
	Separated	0	2 (0.8)	2 (0.4)		
	Widowed	22 (8.8)	23 (9.2)	45 (9)		
4.	Family Type					
	Nuclear	103 (41.2)	96 (38.4)	199 (39.8)	0.79	0.67
	Joint	145 (58)	153 (61.2)	298 (59.6)		
	Others	2 (0.8)	1 (0.4)	3 (0.6)		
5.	Socioeconomic class (Kuppuswamy)					
	Upper (I)	6 (2.4)	12 (4.8)	18 (3.6)		
	Upper Middle (II)	101 (40.4)	111 (44.4)	212 (42.4)	3.99	0.40
	Lower middle (III)	71 (28.4)	68 (27.2)	139 (27.8)		
	Upper Lower (IV)	70 (28.0)	58 (23.2)	128 (25.6)		
	Lower (V)	2 (0.8)	1 (0.4)	3 (0.6)		
6.	Medication adherence					
	Low	102 (40.8)	104 (41.6)	206 (41.2)	0.24	
	Medium	69 (27.6)	72 (28.8)	141 (28.2)		0.88
	High	79 (31.6)	74 (29.6)	153 (30.6)		
7.	Number of prescribed Medication					
	1	0	3 (1.2)	3 (0.6)	3.15	
	2–3	102 (40.8)	99 (39.6)	201 (40.2)		0.36
	4–5	125 (50)	127 (50.8)	252 (50.4)		
	>5	23 (9.2)	21 (8.4)	44 (8.8)		
8.	Family h/o early CVD					
	Yes	74 (29.6)	66 (26.4)	140 (28)	0.63	
	No	176 (70.4)	184 (73.6)	360 (72)		0.42
9.	Duration of treatment					
	<5 years	201 (80.4)	191 (76.4)	392 (78.4)	1.99	
	5–10 years	34 (13.6)	36 (14.4)	70 (14)		0.36
	>10 years.	15 (6)	23 (9.2)	38 (7.6)		

Figures in parentheses are percentages

Results also revealed that both the groups were comparable at baseline, in terms of the number of prescribed medicines, illness duration and medication scores (Table 4).

**Table 4:** Mean difference in the number of prescribed medicines, duration of illness and medication adherence scores at baseline among intervention and comparison group subjects enrolled for secondary prevention of CVDs

S.No	Variable	Intervention group *n* = 250	Comparison group *n* = 250	Mean difference	95%CIof mean difference	t statistics	*P* value
1.	Mean number of prescribed medicines (±1 SD)	3.81 ± 1.15	3.78 ± 1.14	0.03	−0.16,0.23	0.31	0.74
2.	Mean duration of illness (years)	3.16 ± 3.42	3.42 ± 4.24	0.25	−0.97,0.45	0.71	0.47
3.	Mean medication adherence scores (MMAS-8)	6.12 ± 1.91	6.12 ± 1.93	0.01	−0.31,0.35	0.11	0.82

Follow up of the patients enrolled for secondary prevention of CVDs was done at 1 year to assess the effect of intervention on medication adherence. The proportion of subjects available at 1st, 3rd and 6th month follow up were 99.8, 96 and 94.4% respectively. However the number decrased to 87.6% (438) for final follow up at 1 year. During the course of study 1.2% of participants died because of all cause mortality. The data presented in Table 5 shows that there was a significant increases in the mean medication adherence scores (as per MMAS-8 scale) from 6.12 at baseline to 7.60 at follow up with the mean change of 1.48 among subjects in the intervention group (*p* < .01). Change in the medication adherence score in the intervention group also showed large effect size. Whereas in the comparison group the mean change between baseline and post intervention medication adherence was not statistically significant.

**Table 5:** Mean change in medication adherence scores at baseline and at 1 year follow up among intervention and comparison group subjects enrolled for secondary prevention of CVDs

S.No	Group	Baseline	Post intervention	Mean change (95% CI)	% Change	t statistic	*P* value	Cohen’s d
		Mean ± 1SD	Mean ± 1SD					
1.	Intervention group (*n* = 228)	6.12 ± 1.91	7.60 ± 1.00	1.48 (1.72,1.22)	24.18	11.66	0.001	0.97
2.	Comparison group (*n* = 210)	6.12 ± 1.93	5.96 ± 1.82	0.16 (0.10,-0.43)	−2.61	−1.17	0.24	0.08

Mean adherence scores after 1 year follow up was significantly higher in the intervention group (7.60) as compared to comparison group (5.96) with the mean difference of 1.63 (*p* < .01) and large effect size of 1.1 (Table 6).

**Table 6:** Mean difference in the post intervention medication adherence scores in intervention and comparison group subjects enrolled for secondary prevention of CVDs at 1 year follow up

S.No	Medication adherence	Mean	SD	Mean difference	% difference	*t* statistics	*P* value	Cohen’s d
1.	Intervention group (*n* = 228)	7.60	1.00	1.63 (1.36,1.91)	24.18%	11.72	<0.001	1.1
2.	Comparison group (*n* = 210)	5.96	1.82					

Thus the nurse led intervention was effective in risk modification and improving medication adherence for primary and secondary prevention of CVDs respectively.

## Discussion

Task shifting interventions to optimally utilize existing health care workforce can be one of the best available options for implementing CVD prevention programs in view of the current HRH deficit and increasing prevalence of CVDs. Task shifting can be done with various categories of workers e.g. nurses, pharmacists, community health workers etc. Various studies from the LMICs have demonstrated that community health workers can be trained and effectively utilized for CVD risk management [35–38], however to the best of our knowledge there are no studies of nurses being involved for CVD risk assessment and management from India and most of the evidence of nurses doing this task is available from the Western world [17, 39]. The nurses were chosen for this task shifting intervention in the present study as they are better qualified and one of largest category of workforce in any health care institution, yet at present they are underused and underutilized for the task of cardiovascular risk assessment and management in India.

Research design adopted for the study was quasi experimental with pre and post test. Although randomized controlled trial is an ideal study design but the same was not chosen because of ethical constraints. As the routine CVD risk screening is not done in the OPDs of the selected hospital so it was found unethical to deny the patient of risk communication and advices about lifestyle modification after assessing the risk. Hence no control group was taken. However there was a comparison group for the secondary prevention participants, as the patients were visiting cardiology OPDs because of the presence of coronary artery disease so it was expected that the patients might have got some education about lifestyle modification and medication adherence from different health care professionals involved in the care. So the comparison group was taken to assess the effect of intervention.

Task shifting intervention in the present study was limited to risk assessment and communication only. However cardiovascular risk management in addition to advices about lifestyle modification also requires the prescription of medications for risk reduction. As nurses in India are not authorized to prescribe medicines so prescription of medications by nurses was not included in the study. Similar approach was adopted in the study (RAPCAPS) by Joshi R et al. [40] where the cardiovascular risk factor screening was done by non physician health worker and the patient had to take medication prescription from the physician during second consultation. Moreover our study was planned in tertiary health care hospital where there is availability of medical experts, so the need for prescription of medicines by nurses was not justified in the study.

Intervention in the present study included CVD risk assessment and communication by trained nurses which was followed by three telephonic reminders to reinforce risk reduction at 1st, 3rd and 6th month. Cicolini G et al. [41] found that nurse led telephonic and email reminders significantly improved CVD risk factors and followed the similar follow up schedule.

Follow up of the participants at 1 year revealed that 35.2% of the subjects shifted down to low risk category from moderate, high and very high risk categories. Nearly the same percentage of participants (37.2%) in the ANCHOR study also shifted to lower risk category after 1 year of intervention. However 5.8% of the participants moved to the higher risk category among all risk category groups in our study whereas in ANCHOR the 9.9% moved to the high risk category. The difference could be because we included all the risk category paticipants in our study whereas in ANCHOR study only moderate and high group participants were included as a primary prevention cohort [42]. Another study by Tiessen et al. also observed significant reductions in SCORE CVD 10 year risk with the nurse led intervention in Europe [17].

Interventions for the secondary prevention included risk communication and advices about lifestyle modification. Since medication adherence is one of the important and largest component of medical treatment so it was chosen as an outcome of interest for secondary prevention of CVDs. Adherence to medication is one of the main determining factor of treatment success. Intervention by the nurses in the present study was found to be effective in improving medication adherence of the subjects in the intervention group. Although there is lack of evidence related to nurse led intervention to improve adherence in India, but the health workers involvement was found to be effective in improving adherence among acute coronary syndrome patients recruited from 14 hospitals of India [43]. Studies done in other parts of the World also revealed the effectiveness of nurse led intervention for secondary prevention of CVDs. Clark et al. in their systematic review and meta analysis found that secondary CVD prevention programs are effective in reduction of all-cause mortality and acute myocardial infarction. Nearly half (45%) of the randomized controlled trial included in this meta-analysis were nurse led or nurse managed [44].

Results of the present study revealed that mean adherence score at 1 year follow up was significantly higher in the intervention group (7.60) as compared to comparison group (5.96) with the mean difference of 1.63 and a large effect size of 1.1. Kripalani S in their systematic review also reported that the effect size of informational trial to improve adherence ranges from 0.35 to 1.13 [45]. Jeffery RA et al. in their systematic review highlighted that educational intervention showed significant improvement in medication adherence as compared to usual care [46]. The findings are consistent with the our study results where intervention group showed significantly higher medication adherence scores than comparison group.

So the present study has demonstrated that of CVD risk assessment and communication by nurses is effective in risk modification and improving medication adherence for primary and secondary prevention of CVDs respectively.

The study results imply that policy makers and institute authorities can assign the task of CV risk management to nurses as a policy decision by including these in their job description. Nurses are presently underutilized in the area of CVD risk assessment and management. Using task shifting approach of cardiovascular risk assessment by nurses would help doctors to do tasks that require high level of professional training and skills.

So nurses can play a bigger role in CVD prevention and this will optimize the use of existing human resource for health without putting extra financial burden of recruiting more health workforce. Cardiovascular risk assessment and management by nurses can also be seen as a sustainable and cost effective option for management of CVDs in LMICs like India.

The main strength of the study is that existing manpower was being trained and utilized for risk assessment and communication so there was no additional cost of human resource in the study. Therefore the intervention may be suitable for long term sustainability.

Study also had certain limitations: (1) Cost effectiveness analysis was not done so it is recommended that future studies should also incorporate cost effectiveness analysis. (2) The study is being conducted in a tertiary health care setting which may limit the generalization of the findings.

## Conclusion

The study concludes that nurses can be trained in CVD risk assessment and management. Nurse led intervention was effective in CVD risk modification for primary prevention of CVDs and also improved medication adherence for secondary prevention of CVDs.

## Data Availability

The dataset analysed for the current study are not publicly available due to the ethical restrictions related to the consent given by the participants at the time of study commencement. An ethically compliant dataset may be made available by the corresponding author on reasonable request.

## Appendix

**Table 7:** Shift in the WHO/ISH risk category after 1 year follow up among subjects enrolled for primary prevention of CVDs in a tertiary health care hospital in Chandigarh

S.No	CVD risk category Baseline	CVD risk at follow up					Total
		<10%	10–20%	20–30%	30–40%	> 40%	
1.	Low risk (<10%)	202 (92.7)	13 (6)	2 (0.9)	1 (0.5)	0	218 (60.6)
2.	Moderate Risk (10–20%)	28 (35)	49 (61.3)	3 (3.8)	0	0	80 (22.2)
3.	High risk (20–30%)	16 (36.4)	20 (45.5)	6 (13.6)	1 (2.3)	1 (2.3)	44 (12.2)
4.	Very high risk (30–40%)	5 (55.6)	2 (22.2)	1 (11.1)	1 (11.1)	0	9 (2.5)
5.	Very high risk (>40%)	1 (11.1)	6 (66.7)	1 (11.1)	1 (11.1)	0	9 (2.5)
Total		252 (70)	90 (25)	13 (3.6)	4 (1.1)	1 (0.3)	360

Mc Nemar <.01

Majority of the subjects (92.7%) in the low risk category remained in the same risk category at 1 year follow up. Among subjects in the moderate risk category nearly two third (61.3%) remained in the same category, however approximately one third (35%) moved to the low risk category. In the high risk group 36.4% subjects shifted risk down two categories, from high to low and 45.5% subjects shifted one category from high to moderate. There were significantly higher proportion of participants in the low risk category and lower proportion in high risk category at 1 year follow up in comparison to baseline assessment.(Mc Nemar <.01)

## Abbreviations

CABG: Coronary artery bypass graft surgery
CVD: Cardiovascular disease
FTND: Fagerstrom test for nicotine dependence
GKCSAF: Gap Kalamazoo communication skill assessment form
HTN: Hypertension
ISH: International society for hypertension
JNC: Joint National Committee
LMIC: Low and middle income countries
MMAS: Morisky Medication Adherence Scale
NCD: Non communicable disease
NPHW: Non Physician Health Worker
OPD: Outpatient Department
PTCA: Percutaneous transluminal coronary angioplasty
SBP: Systolic blood pressure
SEAR: South East Asian Region
WHO: World health organization
